# Taxonomic review of the *postica*-group of *Fannia* Robineau-Desvoidy (Diptera, Fanniidae) from China, with the description of one new species

**DOI:** 10.3897/zookeys.112.947

**Published:** 2011-06-24

**Authors:** Ming-fu Wang, Kai Li, Dong Zhang

**Affiliations:** 1 Institute of Entomology, Shenyang Normal University, Shenyang 110034, Liaoning, P. R. China; 2 College of Biological Sciences and Biotechnology, Beijing Forestry University, Beijing 100083, P. R. China

**Keywords:** Diptera, Fanniidae, *Fannia*, *Fannia postica*-group, new species

## Abstract

The Chinese fauna of the *Fannia postica*-group Chillcott (1961) is reviewed, the diagnostic features of this group are redefined, one new species, *Fannia nudifemorata* **sp. n.**, is described, and a key to the males of nine known species is given. One new junior synonym, *Fannia tigripeda* Xue, Wang&Li, **syn. n.** for *Fannia stigi* Rognes is established. To facilitate comparisons of the species, *Fannia aethiops* Malloch, *Fannia ardua* Nishida, *Fannia discoculea* Xue, *Fannia ringdahlana* Collin, *Fannia postica* (Stein), *Fannia spathiophora* Malloch, and *Fannia stigi* Rognes are redescribed and illustrated. The geographic distribution of the known Chinese species is updated.

## Introduction

The *Fannia postica*-group was originally established in the genus *Fannia* Robineau-Desvoidy by Chillcott (1961), consisting of two subgroups, the *Fannia postica*-subgroup and the *Fannia spathiophora-*subgroup. Fifteen species were included from the Holarctic Region (Chillcott 1961). Since then, (Nishida (1975, 1976), Rognes (1982), and Xue et al. (2001) have studied the *Fannia postica*-group from Japan, Europe, and China, respectively, forming a sound basis for further research on this group. Rozkošný et al.(1997) listed nine European species of the *Fannia postica*-group and transferred *Fannia ringdahlana* Collin from the *Fannia spathiophora*-subgroup to the *Fannia postica*-subgroup. Gregor and Rozkošný (2005) described an additional new species of the *Fannia postica*-group from Slovakia. So far, twenty-two species of the *Fannia postica*-group are known worldwide, of which eight species belong to the *Fannia postica*-subgroup and the others to the *Fannia spathiophora*-subgroup.

Before the present contribution, eight species of the *Fannia postica-*group were known in China (Nishida 1975, Fan 1992, Xue and Wang 1998, Xue et al. 2001, Wang and Xue 2002, Wu and Wang 2002, Su and Wang 2004, Wang et al. 2006). In recent years we have been engaged in faunal studies of this group of flies in China, and have found one further undescribed species from the Tibetan Plateau.The purpose of this paper is to review the *Fannia postica-*group, describe one new species, and provide a key to the known Chinese species. Based upon the morphological characters, we also discuss the diagnostic characters of this group.

## Materials and methods

The morphological terminology follows McAlpine (1981), except that we follow Stuckenberg (1999) in using “postpedicel” for first antennal flagellomere. Absolute measurements are used for body length in millimetres (mm). Abbreviations used for characters include: *a* = anterior seta, *acr* = acrostichal seta, *ad* = anterodorsal seta, *av* = anteroventral seta, *d* = dorsal seta, *dc* = dorsocentral seta, *ia* = intra-alar seta, *p* = posterior seta, *pd* = posterodorsal seta, *pra* = prealar seta, and *pv* = posteroventral seta.

Our study of the *Fannia postica*-group was based on an examination of specimens of *Fannia postica*, *Fannia ringdahlana*, *Fannia aethiops*, *Fannia ardua*, and *Fannia spathiophora*, and the holotypes of *Fannia discoculea*, *Fannia nudifemorata* sp. n., and *Fannia stigi*. Data on *Fannia coculea* Nishida was taken from Nishida (1975). All specimens studied in this paper, including the types of new species, are deposited in the Institute of Entomology, Shenyang Normal University, Shenyang.

## Taxonomic account

### Fannia postica -group

Fannia postica -group: Chillcott, 1961: 101, 222; Rozkošný et al. 1997: 48.

#### Diagnosis.

Each tibia with at most one seta on each surface; mid first tarsomere usually with basal tooth-like spines or clustered hairs on ventral surface; hind femur usually with one or numerous *av*; hind coxa bare on posterior surface (except *Fannia discoculea* and *Fannia coculea*); lower calypter at least leaf-like, otherwise lower calypter projecting beyond upper one; *pra* usually 2; presutural *acr* usually biserial; katepisternum without ventral spines; male cercal plate longish, the median part distinctly swollen in ventral view, the median part curving anteriorly and the apex curving posteriorly in lateral view. For detailed descriptions of the adults, see Chillcott (1961: 124).

Included species: *Fannia postica*-subgroup: *Fannia brevicauda* Chillcott, *Fannia discoculea*, *Fannia enigmata* Chillcott, *Fannia flavibasis* (Stein), *Fannia multisetosa* Chillcott, *Fannia postica*, *Fannia ringdahlana*, *Fannia sequoiae* Chillcott; *Fannia spathiophora*-subgroup: *Fannia aethiops*, *Fannia ardua*, *Fannia bigelowi* Chillcott, *Fannia brooksi* Chillcott, *Fannia coculea*, *Fannia gotlandica* Ringdahl, *Fannia nudifemorata* sp. n., *Fannia scyphocerca* Chillcott, *Fannia slovaca* Gregor & Rozkošný, *Fannia spathiophora*, *Fannia stigi*, *Fannia tundrarum* Chillcott, *Fannia umbratica* Collin, *Fannia umbrosa* (Stein).

#### Key to males of the known Chinese species of the *Fannia postica*-group

**Table:** 

1	Hind femur at least with 2 *av* in distal half (*postica*-subgroup)	2
–	Hind femur only with 1 *av* in distal half (*spathiophora*-subgroup)	4
2	*Pra* 2; hind coxa bare on posterior surface	3
–	*Pra* 1; hind coxa with hairs on posterior surface	*Fannia discoculea*
3	Mid first tarsomere with basal tooth-like spines on ventral surface; hind femur only with 2 *av* in distal half; calypters blackish	*Fannia ringdahlana*
–	Mid first tarsomere without basal tooth-like spines on ventral surface; hind femur with 4–6 *av* in distal half; calypters yellow	*Fannia postica*
4	Hind coxa bare on posterior surface	5
–	Hind coxa with hairs on posterior surface; *pra* 2 (rarely 3); frontal setae 7–9; mid first tarsomere with basal tooth-like spines on ventral surface	*Fannia coculea*
5	Fore tibia with 7–9 long and fine *pv* hairs	*Fannia spathiophora*
–	Fore tibia without such hairs	6
6	Hind femur with *pv* in distal 1/3	7
–	Hind femur without distinct *pv* in distal 1/3; haltere dark brown	*Fannia nudifemorata*
7	Hind femur with 4 or 5 *pv* in distal half	8
–	Hind femur only with 2 or 3 *pv* in distal half; abdominal tergites 2–4 each with a median black stripe	*Fannia aethiops*
8	The median part of frons about as wide as anterior ocellus; frontal setae 6; each abdominal tergite with a triangular mark	*Fannia ardua*
–	The median part of frons about 2.5 times as wide as anterior ocellus; frontal setae 8 or 9; each abdominal tergite with a median black stripe	*Fannia stigi*

### Catalogue of known Chinese species of the *Fannia postica*-group and description of one new taxon

#### Fannia discoculea

Xue in Xue & Wang, 1998

http://species-id.net/wiki/Fannia_discoculea

Figs 1–3

Fannia discoculea Xue in Xue and Wang 1998: 822–824; Wang and Xue 2002: 56; Su and Wang 2004: 111.

##### Description.

MALE.Body length 3.5 mm. Eye bare; fronto-orbital plate and parafacial with greyish-white pruinosity; the median part of frons about 1.5 times as wide as anterior ocellus, fronto-orbital plate adjoined in upper half, frontal setae 5, situated in the lower 2/3 of frons, orbital setae absent; parafacial bare and narrow, about half as wide as postpedicel width at middle part; antenna black, postpedicel 1.5 times as long as wide, arista black, distinctly swollen in basal 1/4, haired, the longest hair about equal to aristal base; epistoma not projecting beyond vibrissal angle, vibrissal angle behind frontal angle in profile; gena and genal dilation with black hairs, upper margin of gena without upcurved setae; proboscis short, prementum slightly shining, with greyish-brown pruinosity, palpus black, slightly longer than prementum. Thorax ground-colour black, with thin greyish-brown pruinosity, scutum without stripes; presutural *acr* biserial, only prescutellar pairs slightly stout, *dc* 2+3, *ia* 0+2, *pra* 1, notopleuron without setulae; basisternum, proepisternum, anepimeron, meron and katepimeron bare; katepisternal setae 1:1, katepisternum without ventral spines; calypters yellowish, lower one slightly projecting beyond upper one. Wing brownish; costal spine inconspicuous; vein Sc curved bow-like; node of Rs bare on ventral and dorsal surfaces; crossveins without obvious cloud; haltere yellow. Legs entirely black; fore tibia without *ad* and median *p*; mid coxa without any hooked spines or spine-like setae on lower and outer margins, mid femur with 15 *av*, becoming gradually shorter towards apex, *pv* row distinct in basal 2/3, *p* row stout in distal part, mid tibia slightly swollen in distal part, with 1 *ad*, 1 *pd* and numerous hairs on ventral surface, the longest one about equal to mid tibia width, mid first tarsomere without basal tooth-like spines on ventral surface, only with a cluster of hairs; hind coxa with hairs on posterior surface, hind femur with 5 *av* and 2 *ad* in distal half, 2 long *pv* in distal 1/4, the longer one slightly longer than hind femoral width, hind tibia with 1 *av*, 1 *ad* and 1 *d*. Abdomen oval, depressed and flattened, ground-colour black, with thin greyish-brown pollinosity; syntergite 1+2 to tergite 4 each with a black triangular mark at middle; sternite 1 with hairs, sternite 5 broad; cercal plate straight in apex, bare on ventral surface, median part distinctly broad; bacilloform process U-shaped in ventral view, ring-like in lateral view.

##### Material examined.

Holotype ♂: China: Xinjiang: Jakesi, 43°49’12”N, 81°07’12”E, 6.VIII.1957, Coll. G. Wang.

##### Distribution.

China (Xinjiang).

**Figures 1–3.**
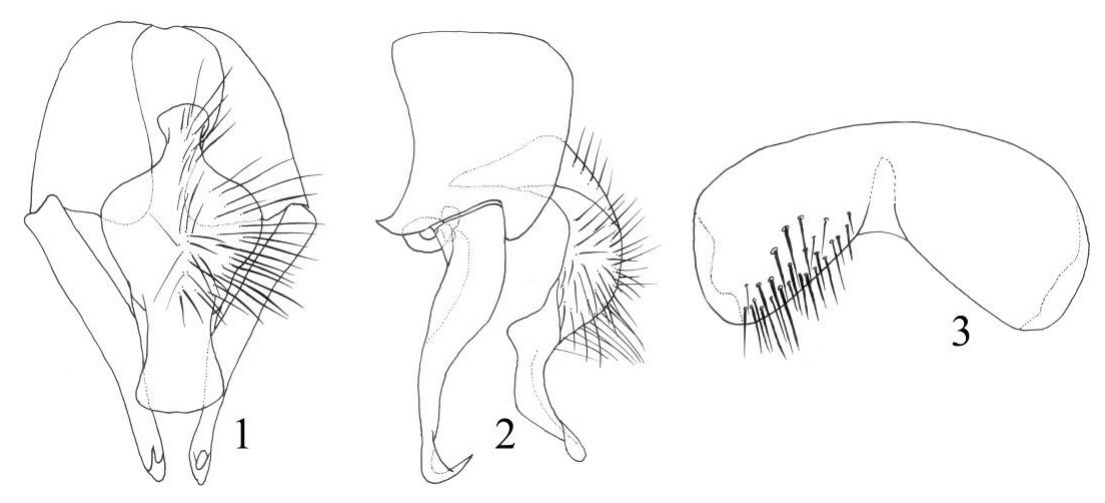
*Fannia discoculea* Xue, 1998 (male) **1** terminalia, ventral view **2** terminalia, lateral view 3 sternite 5.

#### Fannia postica

(Stein, 1895)

http://species-id.net/wiki/Fannia_postica

Figs 4–6

Homalomyia postica Stein 1895: 89.
Fannia postica (Stein): Hennig 1955: 72; Chillcott 1961: 103; Pont 1986: 53; Xue and Wang 1998: 819; Wang and Xue 2002: 57; Su and Wang 2004: 111; Wang et al. 2006: 555.

##### Description.

MALE. Body length 4.0 mm. Eye bare, facets slightly enlarged on anterior margin in upper part; fronto-orbital plate, parafacial and gena with densely grey pruinosity; the median part of frons about 1.5 times as wide as anterior ocellus, as wide as 2/3 of postpedicel, frontal vitta black, linear at middle, frontal setae 6 or 7, situated in the lower 3/4 of frons, orbital setae absent; postocular setulae short, in one row, regularly placed; parafacial bare, about half as wide as postpedicel width at middle; antenna black, postpedicel 2.0 times as long as wide, arista black, distinctly swollen in basal part, ciliated, the longest hair about equal to aristal base; epistoma not projecting beyond vibrissal angle, vibrissal angle behind frontal angle in profile; gena and genal dilation with black hairs; genal height about 1/10 of eye height, upper margin of gena without upcurved setae; proboscis short, prementum with thin greyish-yellow pruinosity, its length 1.8–2.0 times as long as its width, palpus black, claviform, about as long as prementum. Thorax ground-colour black, with dark brown pruinosity, scutum without stripes; presutural *acr* biserial, only prescutellar pairs slightly stout, the distance between two *acr* rows slightly narrower than the distance between *acr* row and *dc* row, *dc* 2+3, *ia* 0+2, *pra* 2, the anterior one about 1/2 length of posterior notopleural seta, notopleuron without setulae; basisternum, proepisternum, anepimeron, meron and katepimeron bare; katepisternal setae 1:1, katepisternum without ventral spines; anterior spiracles yellowish, the posterior ones brownish-yellow; calypters yellow, lower one slightly projecting beyond upper one. Wing brownish; veins brown, tegula dark brown, basicosta brownish-yellow, costal spine inconspicuous; vein Sc curved bow-like; node of Rs bare on ventral and dorsal surfaces; veins R4+5 and M parallel to each other distally; crossveins without obvious cloud; haltere brownish-yellow. Legs entirely black; fore tibia without median *p*, fore first tarsomere with 2 or 3 longish basal setae on ventral surface; mid coxa without any hooked spines or spine-like setae on lower and outer margins, mid femur with sparse and long *av* row in basal half, becoming gradually shorter towards apex, comb-like in basal 1/3, *pv* row complete, becoming gradually shorter towards apex, *p* row complete, mid tibia swollen in distal half, with 1 *ad* and 1 *pd* in distal half, with numerous hairs on ventral surface, the longest ones about 3/4 of mid tibia width, mid first tarsomere without basal tooth-like spines on ventral surface; hind coxa bare on posterior surface, hind femur with short *av* row in basal half, becoming gradually longer towards apex, 4–6 long and stout *av* in distal half, *pv* row inconspicuous in basal half, 4 or 5 stout *pv* in distal half, hind tibia with 1 median *av*, 1 *ad* and 1 *d*. Abdomen oval, depressed and flattened, ground-colour black, with greyish-brown pollinosity; syntergite 1+2 to tergite 4 each with dark triangular mark at middle, tergite 5 only with narrow stripe at middle; sternite 1 bare.

##### Material examined.

China: Heilongjiang: 2 ♂, Xilinji, 53°28'48"N, 122°22'12"E, 19.VI.1986, Coll. C.Y. Cui.

##### Distribution.

China (Heilongjiang), Europe, North America.

**Figures 4–6.**
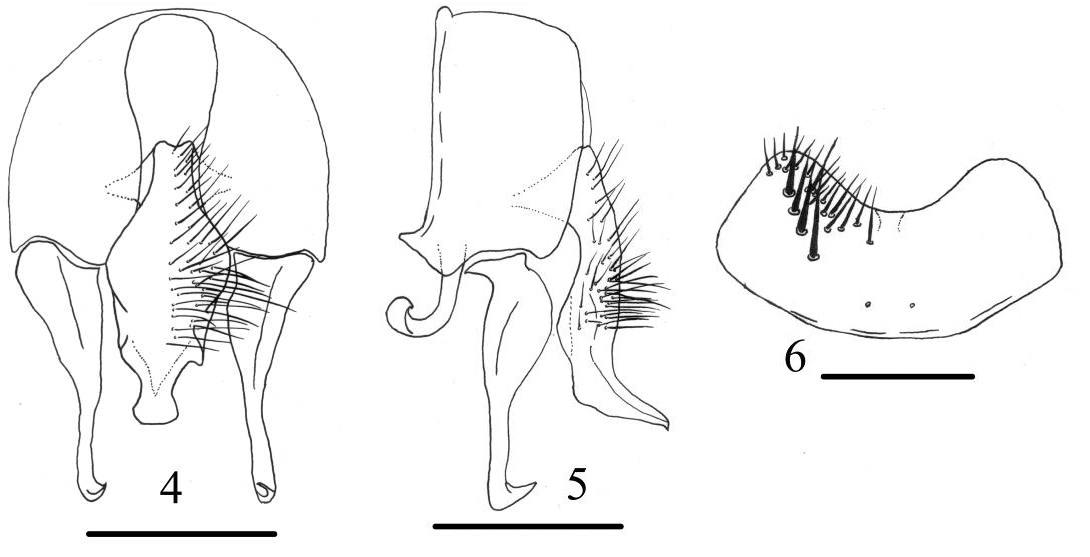
*Fannia postica* (Stein,1895) (male) **4** terminalia, ventral view **5** terminalia, lateral view 6 sternite 5, scale bar = 0.25mm.

#### Fannia ringdahlana

Collin, 1939

http://species-id.net/wiki/Fannia_ringdahlana

Figs 7–9

Fannia.ringdahlana Collin 1939: 14; Nishida 1975: 378; Fan 1992: 216; Xue and Wang 1998: 815; Wang and Xue 2002: 57; Su and Wang 2004: 112; Wu and Wang 2002: 563; Wang et al. 2004: 34; Wang et al. 2006: 555.

##### Description.

MALE. Body length 4.0–4.8 mm. Eye with short and brownish hairs, facets slightly enlarged on anterior margin in upper part; fronto-orbital plate with brownish-grey pruinosity in upper half, lower half of fronto-orbital plate and parafacial with densely silvery-grey pruinosity; the median part of frons about 1.5 times as wide as anterior ocellus, 2/3 as wide as postpedicel, frontal vitta black, linear at middle part, frontal setae 11 or 12, nearly reaching ocellar triangle, orbital setae absent; postocular setulae in one row, 4 or 5 ones long and fine in vertex, anteriorly curved, others short, regularly placed; parafacial bare, about1/2 as wide as postpedicel width at middle part; antenna black, postpedicel about 1.5 times as long as wide, arista black, distinctly swollen in basal 1/3, ciliated, the longest hairs slightly shorter than aristal base; epistoma not projecting beyond vibrissal angle, vibrissal angle behind frontal angle in profile; gena and genal dilation with black hairs; genal height about 1/10 of eye height, upper margin of gena without upcurved setae; proboscis short, prementum slightly shining, with thin greyish-yellow pruinosity, its length 1.5 times as long as its width, palpus black, claviform, slightly longer than prementum. Thorax ground-colour black, with dark brown pruinosity, scutum without stripes; presutural *acr* biserial, only prescutellar pairs slightly stout, the distance between two *acr* rows slightly narrower than the distance between *acr* row and *dc* row, *dc* 2+3, *ia* 0+2, *pra* 2, the anterior one about 3/5 of the length of posterior notopleural seta, notopleuron without setulae; basisternum, proepisternum, anepimeron, meron and katepimeron bare; katepisternal setae 1:1, katepisternum without ventral spines; spiracles dark brown; calypters dark brown, blackish-brown on the margin, lower one slightly projecting beyond upper one. Wing dark brown; veins dark brown, tegula black, basicosta dark brown, costal spine inconspicuous; vein Sc curved bow-like; node of Rs bare on ventral and dorsal surfaces; veins R4+5 and M conspicuously close to each other distally; crossveins without obvious cloud; haltere blackish-brown in base and apex, median part brown. Legs entirely black; fore tibia without *ad* and median *p*, fore first tarsomere with 2 or 3 longish basal setae on ventral surface; mid coxa without any hooked spines or spine-like setae on lower and outer margins, mid femur with sparse and long *av* row in basal half, becoming gradually shorter and denser towards apex, comb-like in basal 1/3, *pv* row complete, long and stout, slightly biserial at middle part, mid tibia distinctly swollen in distal half, with 1 *ad* and 1 *pd* in distal half, with numerous hairs on ventral surface, the longest one about 4/5 of mid tibia width, mid first tarsomere with basal tooth-like spines on ventral surface; hind coxa bare on posterior surface, hind femur with 2 *av* in subapical, 5 *ad* and 2 *d* in distal 1/3, *p* row seta-like in basal half, becoming gradually longer towards apex, 7 or 8 *pv* in distal 1/3, hind tibia with 1 *av*, 1 *ad*, 1 submedian *d* and 1 apical *d*, with numerous erect median setae on posterior surface. Abdomen long, depressed and flattened, ground-colour black, with densely brownish-grey pollinosity; syntergite 1+2 to tergite 4 each with a large triangular mark at middle, tergite 5 with a dark median stripe in basal half, the setae long and stout on the lateral margin of each tergite; sternite 1 with 1–3 fine and long setae on each lateral margin.

##### Material examined.

China: Yunnan: 5 ♂, Xianggelila, Bitahai, 27°48'00"N, 99°54'00"E, 3700m, 2.VII.2006, Coll. M.F. Wang; 1 ♂, same locality and time, Coll. B.F. Wang; 5 ♂, same locality and time, Coll. L. Chang; 1 ♂, Deqin, Mt. Meili, 28°29'24"N, 98°55'48"E, 4000–4200m, 2.VII.2006, Coll. Y. Wang. Shanxi: 1 ♂, Ningwu, Mt. Luya, 38°43'48"N, 111°55'48"E, 12.VI.1987, Coll. M.F. Wang. Jilin: 2 ♂, Mt. Changbai, 42°19'48"N, 127°16'12"E, 18.VII.1988; 2 ♂, Mt. Changbai, Xiaotianchi, 42°34'48"N, 128°18'00"E, 25.VII.1982, Coll. L.Y. Gao. Sichuan: 1 ♂, Daocheng, Kasi, 29°2'24"N, 100°18'36"E, 2750–3000m, 12.VII.2006, Coll. C.T. Zhang; 9 ♂, Jiuzaigou, 33°15'36"N, 103°54'36"E, 2800m, 3.VI.2006, Coll. D. Wang; 3 ♂, same locality, 2.VI.2006, Coll. D. Jing; 2 ♂, same locality, 1.VI.2006, Coll. Y. Zhu.

##### Distribution.

China (Shanxi, Jilin, Sichuan, Yunnan, Taiwan), Japan, Europe.

**Figures 7–9.**
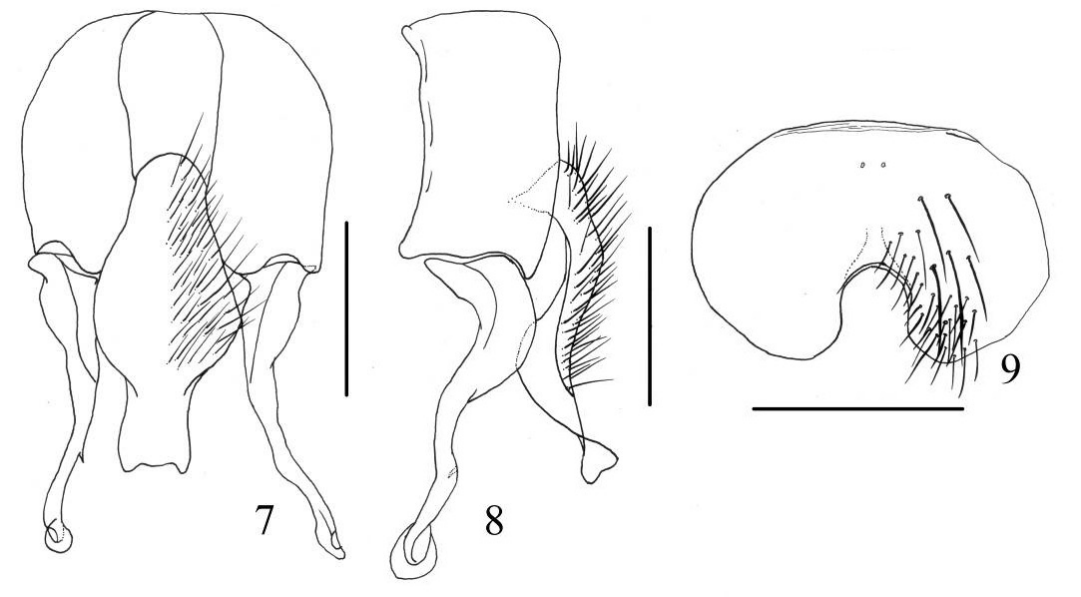
*Fannia ringdahlana* Collin, 1939 (male) **7** terminalia, ventral view, scale bar = 0.25mm **8** terminalia, lateral view, scale bar = 0.25mm **9** sternite 5, scale bar = 0.40mm.

#### Fannia aethiops

Malloch, 1913

http://species-id.net/wiki/Fannia_aethiops

Figs 10–12

Fannia aethiops Malloch 1913: 628; Wang and Xue 1993: 458; Xue and Wang 1998: 818; Wang and Xue 2002: 55.

##### Description.

MALE. Body length 4.5 mm. Eye bare, facets slightly enlarged on anterior margin in upper part; fronto-orbital plate with golden-brown pruinosity in upper half, lower half of fronto-orbital plate and parafacial with densely grey pruinosity; the median part of frons about 1.5 times as wide as anterior ocellus, 3/5 as wide as postpedicel, frontal vitta black, linear in narrowest part, frontal setae 9, nearly reaching ocellar triangle, orbital setae absent; postocular setulae in one row, short, regularly placed; parafacial bare, about as wide as 2/5 of postpedicel width at middle; antenna black, postpedicel broad, about 1.5 times as long as wide, arista black, distinctly swollen in basal part, ciliated, the longest hairs slightly shorter than aristal base; epistoma not projecting beyond vibrissal angle, vibrissal angle behind frontal angle in profile; gena with thin greyish-brown pruinosity, gena and genal dilation with black hairs; genal height about 1/11 of eye height, upper margin of gena without upcurved setae; prementum slightly shining, without distinct pruinosity, its length 1.5 times as long as its width, palpus black, slightly swollen and depressed in apex, about as long as prementum. Thorax ground-colour black, scutum and scutellum with densely brown pruinosity, pleura with thin greyish-brown pruinosity, scutum without distinct stripe; presutural *acr* biserial, only prescutellar pairs slightly stout, the distance between two *acr* rows slightly narrower than the distance between *acr* row and *dc* row, *dc* 2+3, *ia* 0+2, *pra* 2, the anterior one about 1/2 of the length of posterior notopleural seta, notopleuron without setuaae; basisternum, proepisternum, anepimeron, meron and katepimeron bare; katepisternal setae 1:1, katepisternum without ventral spines; spiracles brown; calypters brownish-yellow, lower one slightly projecting beyond upper one. Wing brownish; veins brown, tegula dark brown, basicosta brownish-yellow, costal spine conspicuous, about as long as 2/3 of crossvein r-m; vein Sc curved bow-like; node of Rs bare on ventral and dorsal surfaces; vein R4+5 straight, veins R4+5 and M conspicuously close to each other distally; crossveins without obvious cloud; haltere yellowish-brown. Legs black; fore tibia without median *p*, fore first tarsomere with few longish basal setae on ventral surface; mid coxa without any hooked spines or spine-like setae on lower and outer margins, mid femur with sparse and long *av* row in basal half, becoming gradually shorter and denser towards apex, bare in subapical part, 3 or 4 short setae in apical part, *pv* row complete, long and stout, slightly biserial on middle part, *p* row fine and long, mid tibia distinctly swollen in distal half, with 1 *ad* and 1 *pd* in distal half, with numerous hairs on ventral surface, the longest one about 3/4 of mid tibia width, mid first tarsomere with clustered basal hairs on ventral surface; hind coxa bare on posterior surface, hind femur with 1 stout *av* and 2 or 3 *pv* in subapical part, hind tibia with 1 median *av*, 1 *ad* and 1 *d*. Abdomen long, depressed and flattened, ground-colour black, with densely greyish-brown pollinosity; syntergite 1+2 with broad black stripe on middle, tergites 3 and 4 with narrow black stripe on middle, tergite 5 without stripe; sternite 1 with 1or 2 long setae on each lateral margin.

##### Material examined.

China: Shanxi: 1 ♂, Ningwu, Mt. Luya, 38°43'48"N, 111°55'48"E, 12.VI.1987, Coll. M.F. Wang. Jilin: 1 ♂, Mt. Changbai, 42°19'48"N, 127°16'12"E, 22.VI.1980, Coll. Z.Y. Ma.

##### Distribution.

China (Shanxi, Jilin), North America, Europe.

**Figures 10–12.**
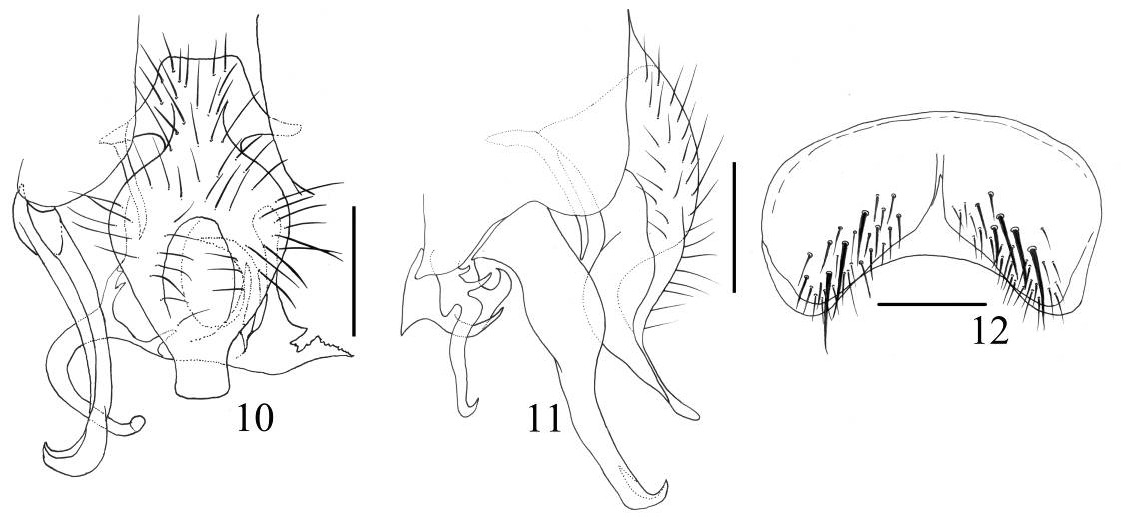
*Fannia aethiops* Malloch, 1913 (male) **10** terminalia, ventral view **11** terminalia, lateral view **12** sternite 5, scale bar = 0.20mm.

#### Fannia ardua

Nishida, 1976

http://species-id.net/wiki/Fannia_ardua

Figs 13–15

Fannia ardua Nishida 1976: 135–137; Wang and Xue 2002: 55; Su and Wang 2004: 110; Wang et al. 2006: 555.

##### Description.

MALE. Body length 4.0 mm. Eye bare, facets slightly enlarged on anterior margin in upper part; fronto-orbital plate with greyish-brown pruinosity in upper half, lower half of fronto-orbital plate and parafacial with silvery-grey pruinosity; the median part of frons about as wide as anterior ocellus, frontal vitta linear at middle, frontal setae 7 or 8, situated in the lower 2/3 of frons, orbital setae absent; postocular setulae in one row, regularly placed; parafacial bare, about 1/3 as wide as postpedicel width at middle; antenna black, postpedicel about 1.2 times as long as wide, arista black, distinctly swollen in basal 1/5, ciliated, the longest hairs about as long as aristal base; epistoma not projecting beyond vibrissal angle, vibrissal angle behind frontal angle in profile; gena and genal dilation with black hairs; genal height about 1/10 of eye height, upper margin of gena without upcurved setae; prementum shining, with thin grey pruinosity, its length 2.0 times as long as its width, palpus black, claviform, slightly longer than prementum. Thorax ground-colour black, with thin greyish-brown pruinosity, scutum without distinct stripe; presutural *acr* biserial, only prescutellar pairs slightly stout, the distance between two *acr* rows slightly narrower than the distance between *acr* row and *dc* row, *dc* 2+3, *ia* 0+2, *pra* 2, the anterior one about 1/2 as long as posterior notopleural seta, notopleuron without setulae; basisternum, proepisternum, anepimeron, meron and katepimeron bare; katepisternal setae 1:1, katepisternum without ventral spines; spiracles brown; calypters brown, lower one slightly projecting beyond upper one. Wing brownish; veins brown, tegula black, basicosta brown, costal spine inconspicuous; vein Sc curved bow-like; node of Rs bare on ventral and dorsal surfaces; vein R4+5 straight, veins R4+5 and M conspicuously close to each other distally; crossveins without obvious cloud; haltere yellowish-brown. Legs entirely black; fore tibia without *ad* and median *p*; mid coxa without any hooked spines or spine-like setae on lower and outer margins, mid femur narrowed and bare in subapical part, with 6 or 7 sparse and long *av* in basal 2/5, becoming gradually shorter and denser towards apex, *pv* row complete, biserial at middle, *p* row stout, mid tibia distinctly swollen in distal part, with 1 *ad* and 1 *pd* in distal half, with numerous hairs on ventral surface, the longest ones shorter than mid tibia width, mid first tarsomere with 1 tooth-like basal process on ventral surface; hind coxa bare on posterior surface, hind femur with 1 subapical *av*, 1 apical *d*, 1 apical *pd*, 3 *ad* in distal 1/3 and 5 *pv* in distal half, hind tibia with 1 *av*, 1 *ad*, 1 median *d* and 1 apical *d*. Abdomen long, depressed and flattened, ground-colour black, with densely greyish-brown pollinosity; syntergite 1+2 to tergite 4 each with dark triangular mark at middle, tergite 5 only with a dark stripe at middle; sternite 1 with 4 or 5 long setae on each lateral margin.

##### Material examined.

China: Jilin: 1 ♂, Mt. Changbai, 42°19’48”N, 127°16’12”E, 10.VII.1998.

##### Distribution.

China (Jilin), Japan.

**Figures 13–15.**
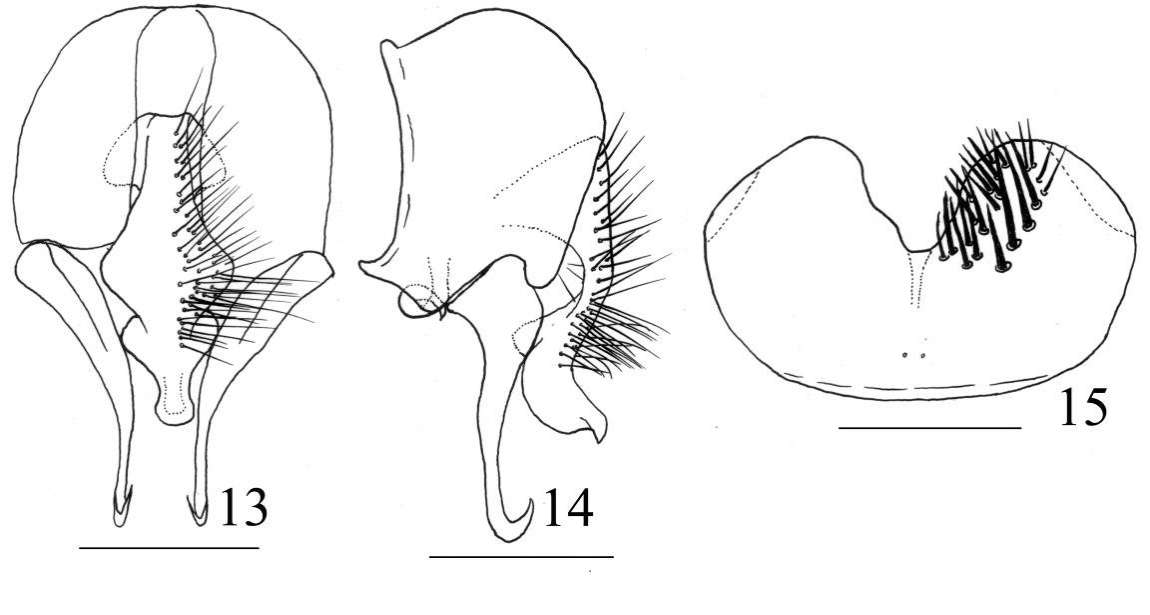
*Fannia ardua* Nishida, 1976 (male) **13** terminalia, ventral view **14** terminalia, lateral view **15** sternite 5, scale bar = 0.25mm.

#### Fannia coculea

Nishida, 1975

http://species-id.net/wiki/Fannia_coculea

Fannia coculea Nishida 1975: 368–370; Xue and Wang 1998: 815; Wang and Xue 2002: 56; Su and Wang 2004: 110.

##### Distribution.

China (Taiwan).

#### Fannia nudifemorata

Wang and Zhang sp. n.

urn:lsid:zoobank.org:act:49E9C8CB-DF77-4B77-AC17-4B3589494995

http://species-id.net/wiki/Fannia_nudifemorata

Figs 16–18 

##### Description.

MALE. Body length 4.5–5.0 mm. Eye bare, facets slightly enlarged on anterior margin in upper part; fronto-orbital plate, parafacial and gena with silvery-grey pruinosity; the median part of frons about 2.0 times as wide as anterior ocellus, about 4/5 of postpedicel, slightly narrower than the distance between two posterior ocelli, frontal vitta black, about as wide as fronto-orbital plate, frontal setae 11–13, situated in the lower 4/5 of frons, the gaps filled with numerous fine setae, orbital setae absent; postocular setulae long and curved anteriorly; parafacial bare, about 2/5 as wide as postpedicel width at middle; antenna black, postpedicel about 1.5 times as long as wide, arista black, distinctly swollen in basal part, ciliated, the longest hairs shorter than aristal base; epistoma not projecting beyond vibrissal angle, vibrissal angle behind frontal angle in profile; gena and genal dilation with black hairs; genal height about 1/9 of eye height, upper margin of gena with 1 or 2 upcurved setae; prementum shining, with thin greyish-yellow pruinosity, its length 2.0 times as long as its width, palpus black, claviform, slightly longer than prementum. Thorax ground-colour black, with brownish-grey pruinosity, scutum without distinct stripe; presutural *acr* biserial, only prescutellar pairs slightly stout, the distance between two *acr* rows about 1/2 of the distance between *acr* row and *dc* row, *dc* 2+3, *ia* 0+2, *pra* 2, the anterior one about 1/2 of the length of posterior notopleural seta, notopleuron without setulae; basisternum, proepisternum, anepimeron, meron and katepimeron bare; katepisternal setae 1:1, katepisternum without ventral spines; spiracles brown; calypters brown, lower one not projecting beyond upper one. Wing brownish; tegula black, basicosta brown, costal spine inconspicuous; vein Sc curved bow-like; node of Rs bare on ventral and dorsal surfaces; vein R4+5 straight, veins R4+5 and M conspicuously close to each other distally; crossveins without obvious cloud; haltere dark brown at apex. Legs entirely black; fore tibia without median *p*, fore first tarsomere with several longish setae on ventral surface; mid coxa without any hooked spines or spine-like setae on lower and outer margins, mid femur with a long and sparse *av* row in basal half, becoming gradually shorter and denser towards apex, *pv* row long and stout, biserial at middle, *p* row complete and long, mid tibia distinctly swollen in distal half, with 1 *ad* and 1 *pd*, with numerous hairs on ventral surface, the longest one slightly shorter than mid tibia width, mid first tarsomere with basal cluster of hairs on ventral surface; hind coxa bare on posterior surface, hind femur with 1 long subapical *av*, 4 or 5 long *ad* in distal 1/3, and 2 or 3 *p* rows in basal 2/3, *pv* row in distal 2/3, hair-like, hind tibia with 1 median *av*, 1 *ad*, 1 *d* and several erect short setae on posterior surface. Abdomen long, depressed and flattened, ground-colour black, with densely bluish-grey pollinosity; syntergite 1+2 to tergite 4 each with a dark triangular mark at middle, tergite 5 only with a dark stripe at middle, each tergite with long setae on lateral part; sternite 1 with 10–12 setae.

Female: Unknown.

##### Material examined.

Holotype, ♂: China: Yunnan: Yulongxueshan, 27°5'24N, 100°15'00"E, 3200m, 24.V.2007, Coll. W.X. Dong. Paratype, 1 ♂, same locality and time, Coll. S.C. Bai.

##### Remarks.

This new species belongs to the *Fannia spathiophora*-subgroup of *Fannia postica*-group. It can easily be separated from its allies by hind femur without stout *pv* and bare from *p* to *pv* surface in distal 1/3. It resembles *Fannia ardua* but differs from the latter in having the median part of frons about 2.0 times as wide as anterior ocellus, frontal setae 11–13, lower calypter not projecting beyond upper one, haltere dark brown at apex. The new species is also similar to *Fannia umbrosa* (Stein, 1895), but differs from it in having hind femur without distinct *pv* row in distal 1/3.

##### Distribution.

China (Yunnan).

**Figures 16–18.**
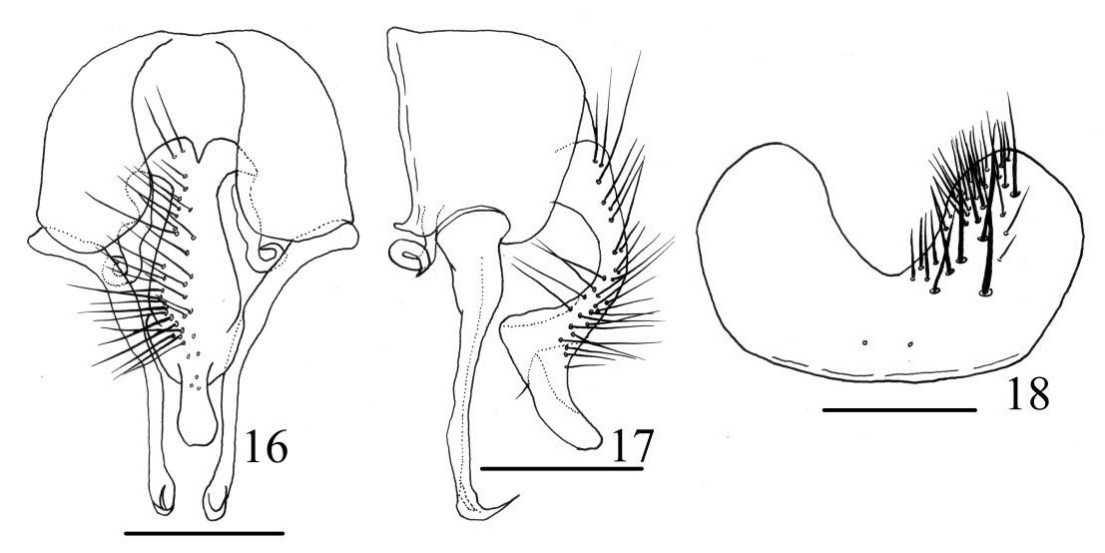
*Fannia nudifemorata* Wang and Zhang, sp. n. (male) **16** terminalia, ventral view **17** terminalia, lateral view **18** sternite 5, scale bar = 0.25mm.

#### Fannia spathiophora

Malloch, 1918

http://species-id.net/wiki/Fannia_spathiophora

Figs 19–21

Fannia spathiophora Malloch 1918: 294; Pont 1986: 56; Fan 1992: 217; Xue and Wang 1998: 815; Wang and Xue 2002: 57; Wu and Wang 2002: 563; Su and Wang 2004: 112; Wang et al. 2004: 34; Wang et al. 2006: 556.

##### Description.

MALE. Body length 4.5–5.0 mm. Eye bare, facets slightly enlarged on anterior margin in upper part; fronto-orbital plate with brownish-grey pruinosity in upper half, lower half of fronto-orbital plate and parafacial with silvery-grey pruinosity; the median part of frons about 1.5 times as wide as anterior ocellus, about 2/3 of pedicel, frontal vitta linear in upper half, frontal setae 7 or 8, situated in the lower 3/4 of frons, orbital setae absent; postocular setulae short, regularly placed; parafacial bare, about 1/3 as wide as postpedicel width at middle; antenna black, postpedicel about 2.0 times as long as wide, arista black, distinctly swollen in basal 1/4, ciliated, the longest hairs slightly shorter than aristal base; epistoma not projecting beyond vibrissal angle, vibrissal angle behind frontal angle in profile; gena and genal dilation with black hairs; genal height about 1/11 of eye height, upper margin of gena without upcurved setae; prementum shining, with thin grey pruinosity, its length 1.5–2.0 times as long as its width, palpus black, claviform, slightly longer than prementum. Thorax ground-colour black, with dense brown pruinosity, scutum without distinct stripe; presutural *acr* biserial, only prescutellar pairs slightly stout, the distance between two *acr* rows slightly narrower than the distance between *acr* row and *dc* row, *dc* 2+3, *ia* 0+2, *pra* 2, the anterior one about 1/2 of the length of posterior notopleural seta, notopleuron without setulae; basisternum, proepisternum, anepimeron, meron and katepimeron bare; katepisternal setae 1:1, katepisternum without ventral spines; spiracles brown; calypters dark brown, lower one projecting beyond upper one. Wing brownish; veins brown, tegula black, basicosta brown, costal spine inconspicuous; vein Sc curved bow-like; node of Rs bare on ventral and dorsal surfaces; vein R4+5 straight, veins R4+5 and M conspicuously close to each other distally; crossveins without obvious cloud; haltere dark brown. Legs black, except knees yellow; fore tibia with 7–9 *pv*; mid femur with long and sparse *av* row in basal half, becoming gradually shorter and denser towards apex, bare in subapical part, 4 or 5 short setae in apical part, *pv* row biserial at middle, *p* row complete, mid tibia distinctly swollen towards apex, with 1 *ad* and 1 *pd* in distal half, 2 long and curved apical hairs on ventral surface, with numerous hairs on ventral surface, the longest ones slightly shorter than mid tibia width, mid first tarsomere with tooth-like basal process on ventral surface; hind coxa bare on posterior surface, hind femur with 1 subapical *av*, *pv* row hair-like in basal half, becoming gradually longer towards apex, 6 or 7 stout and long *pv* in basal 1/3, hind tibia with 1 *av*, 1 *ad* and 1 median *d*. Abdomen long, depressed and flattened, ground-colour black, with dense brownish-grey pollinosity; syntergite 1+2 to tergite 4 each with a dark triangular mark at middle, tergite 5 only with a dark stripe at middle; sternite 1 with 1–3 long setae on each lateral margin.

##### Material examined.

China: Shanxi: 1 ♂, Hunyuan, 39°42'00”N, 113°40'48”E, 12.VII.1985, Coll. M.F. Wang. Liaoning: 2 ♂, Xinbin, Gangshan, 41°43'12"N, 125°01'12"E, -.VI.1981, Coll. Z.Y. Ma; 1 ♂, same locality, 08.IX. 1990; 2 ♂, Benxi, Yanghugou, 41°18'00"N, 123°43'48"E, 01.VII.1993, Coll. Y.S. Cui; 1 ♂, same locality, 01.VII.1993, Coll. C.T. Zhang; 1 ♂, Huanren, 41°16'12"N, 125°21'00"E, 09.VI.1994, Coll. D. Wei; 3 ♂, Qianshan, 41°01'48"N, 123°07'48"E, 25.VI.2007, Coll. M.F. Wang. Jilin: 1 ♂, Baihe, 42°34'48"N, 128°02'24"E, 20.VI.1980, Coll. Z.Y. Ma; 1 ♂, Mt. Changbai, 42°19'48"N, 127°16'12"E, 19.VII.1986; 1 ♂, Mt. Changbai, 42°19'48"N, 127°16'12"E, 15.VII.1990. Heilongjiang: 1 ♂, Guyuan, 50°34'48"N, 123°42'00"E, 26.VI.1980, Coll. C.Y. Cui; 1 ♂, Wuying, 48°06'36"N, 129°14'24"E, 16.VII.1977, Coll. C.Y. Cui; 1 ♂, Bizhou, 51°56'24"N, 124°36'00"E, 13.VII.1980.

##### Distribution.

China (Shanxi, Liaoning, Heilongjiang, Jilin), Japan, Europe, North America.

**Figures 19–21.**
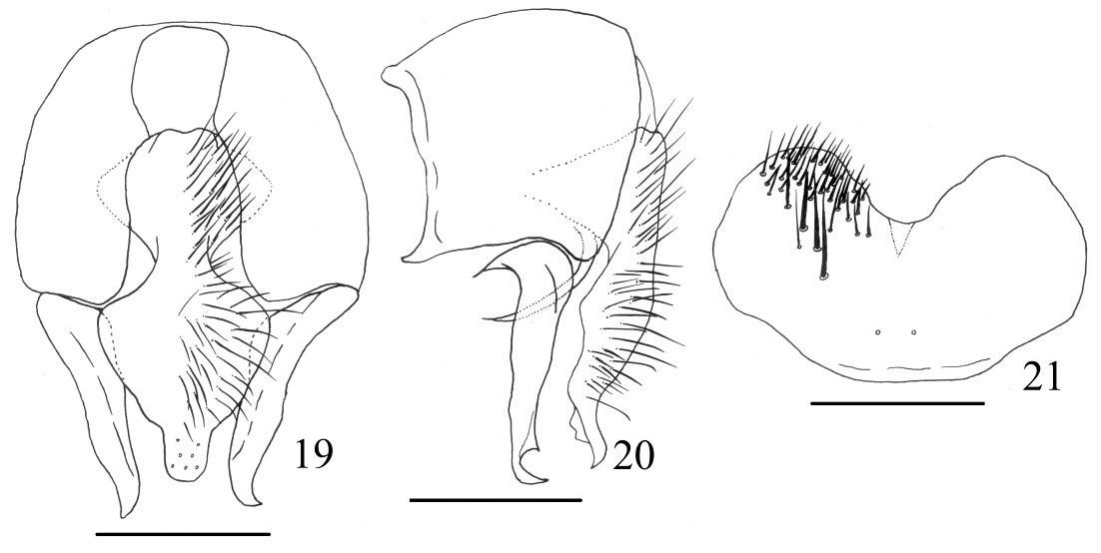
*Fannia spathiophora* Malloch, 1918 (male) **19** terminalia, ventral view **20** terminalia, lateral view **21** sternite 5, scale bar = 0.25mm.

#### Fannia stigi

Rognes, 1982

http://species-id.net/wiki/Fannia_stigi

Figs 22–24

Fannia stigi Rognes, 1982: 325–329.
Fannia tigripeda Xue, Wang and Li 2001: 225–226; Wang and Xue 2002: 57; Su and Wang 2004: 112. syn. nov.

##### Description.

MALE. Body length 4.5–5.0 mm. Eye bare, facets slightly enlarged on anterior margin in upper part; fronto-orbital plate with dark cupreous pruinosity; the median part of frons about 2.5 as wide as anterior ocellus, slightly narrower than postpedicel, frontal vitta black, linear at middle, frontal setae 8 or 9, nearly reaching ocellar triangle; parafacial with silvery-grey pruinosity, slightly narrower than 1/3 of postpedicel width at middle; antenna black, postpedicel about 2.5 times as long as wide, arista distinctly swollen at base, the longest hairs slightly shorter than aristal base; gena and genal dilation with black hairs; genal height about 1/10 of eye height; proboscis short, labella large, the length of prementum 2.5 times as long as its width, palpus black, slightly longer than prementum. Thorax ground-colour black, with thin grey pruinosity, slightly shining, scutum without distinct stripe; presutural *acr* biserial, only prescutellar pairs slightly stout, the distance between two *acr* rows slightly narrower than the distance between *acr* row and *dc* row, *dc* 2+3, *ia* 0+2, *pra* 2, the anterior one about 1/2 the length of posterior notopleural seta, notopleuron without setulae; basisternum, proepisternum, anepimeron, meron and katepimeron bare; katepisternal setae 1:1; spiracles brownish; calypters brownish, lower one not projecting beyond upper one. Wing brownish; tegula black, basicosta brown, costal spine inconspicuous; vein Sc curved bow-like; node of Rs bare on ventral and dorsal surfaces; vein R4+5 straight, veins R4+5 and M parallel to each other distally; crossveins without obvious cloud; haltere dark brown. Legs entirely black; fore tibia without median *p*; mid femur concave at apex on ventral surface, with comb-like *av* row, becoming shorter towards apex, *pv* row distinct in basal 2/3, 1 short and erect *pv* row in subapical part, with 1 complete *p* row, among which subapical 2 stout, mid tibia distinctly swollen in distal half, with 1 *ad* and 1 *pd* in submedian part, with numerous hairs on ventral surface, the longest ones slightly longer than mid tibia width, mid first tarsomere with tooth-like basal process on ventral surface; hind coxa bare on posterior surface, hind femur with 1 short subapical *av*, 4 or 5 long *pv* in distal 1/3, hind tibia with 1 *av*, 1 *ad* and 1 *d* in submedian part. Abdomen long, depressed and flattened, ground-colour black, with brownish-grey pollinosity; syntergite 1+2 to tergite 5 each with a black stripe on middle; sternite 1 with short setae on each lateral margin.

##### Remarks.

Rognes (1982) described *Fannia stigi* as new to science from Norway and Sweden, and also provided detailed description and high-quality illustrations. When re-examined the holotype of *Fannia tigripeda*, we found its morphological characters, especially the male terminalia, is similar with *Fannia stigi*. Whereas, we have not studied the types of *Fannia stigi*, but it is clear from Rognes’s notes and figures on *stigi* that our *tigripeda* is actually his *stigi*. An anonymous reviewer also pointed out the possible synonymy of *Fannia stigi* and *Fannia tigripeda*. We confident that *Fannia tigripeda* is a new junior synonym for *Fannia stigi*.

##### Material examined.

1 ♂: China: Jilin, Mt. Changbai, 42°19'48"N, 127°16'12"E, 1700m, 28.VI.1997, Coll. W.Q. Xue (holotype of *Fannia tigripeda*). Shanxi: 1 ♂, Ningwu, 38°43'48"N, 111°55'48"E, 07.VI.1982, Coll. M.F. Wang.

##### Distribution.

China (Shanxi, Jilin), Norway, Sweden.

## Discussion

Chillcott (1961) revised the Nearctic species of the genus and assigned the 148 Holarctic species to 11 species groups and 15 subgroups, including the *Fannia postica*-group. He recognized that the relationship between the *Fannia postica*-group, *Fannia hirticeps*-group and *Fannia lugubrina*-group was very close (Chillcott 1961). Since Chillcott (1961), Domínguez and Roig-Juñent (2008) proposed a phylogenetic hypothesis of the family Fanniidae, using 151 characters from adult external morphology and female and male terminalia for 78 fanniid species. The analysis, including the *Fannia postica*-subgroup and *Fannia spathiophora-*subgroup, also recovered the paraphyletic of the *Fannia postica*-group, which nested within the *Fannia carbonaria*-group, *Fannia hirticeps*-group and *Fannia lugubrina*-group (Domínguez and Roig-Juñent, 2008). A phylogenetic revision of the genus *Fannia*, from molecular data and more characters of immature stages or adult morphology,at the species-group level is required to establish a more reasonable species-group classification.

After a systematic study of these species and related species, we found that the *Fannia postica*-group can be distinguished from other *Fannia* in having the median part of the male cercal plate distinctly swollen in ventral view, the median part curving anteriorly and the apex curving posteriorly in lateral view. In the course of this study, we also found that a number of characters, including the number of distal *av* on hind femur and the shape of male cercal plate (Chillcott 1961: 101), which have previously been described as being diagnostic characters for the *Fannia postica*-subgroup and *Fannia spathiophora*-subgroup, are variable and unreliable.

**Figures 22–24.**
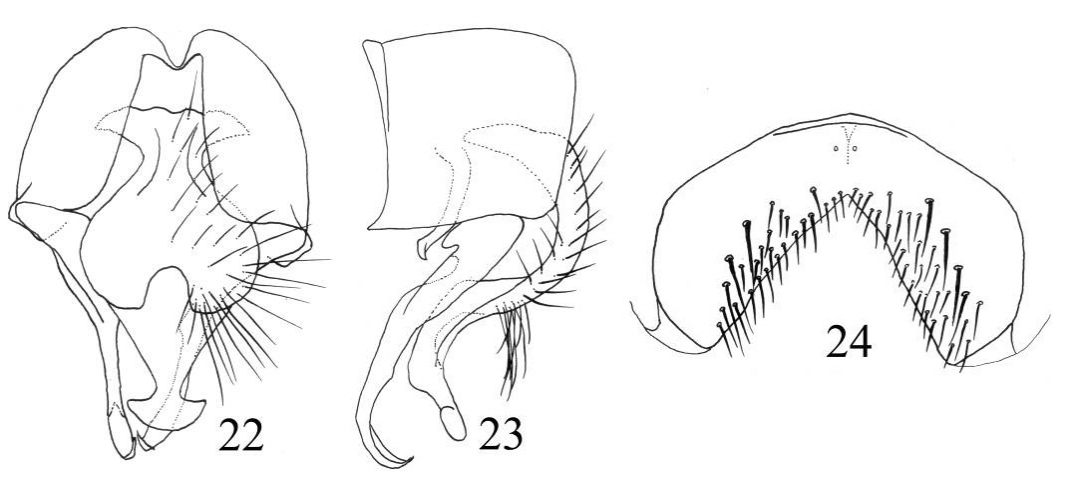
*Fannia stigi* Rognes, 1982 (Holotype of *Fannia tigripeda* Xue, Wang & Li, 2001) (male) **22** terminalia, ventral view **23** terminalia, lateral view **24** sternite 5.

## Supplementary Material

XML Treatment for Fannia postica

XML Treatment for Fannia discoculea

XML Treatment for Fannia postica

XML Treatment for Fannia ringdahlana

XML Treatment for Fannia aethiops

XML Treatment for Fannia ardua

XML Treatment for Fannia coculea

XML Treatment for Fannia nudifemorata

XML Treatment for Fannia spathiophora

XML Treatment for Fannia stigi
